# The post-translational modification of NuMA in cancer cells is a new target for cancer eradication

**DOI:** 10.1038/s41419-025-07868-7

**Published:** 2025-07-18

**Authors:** Malka Cohen-Armon

**Affiliations:** Faculty of Medical and Health Sciences, Gray School of Medicine and Sagol School of Neuroscience, Tel-Aviv University, Tel Aviv, Israel

**Keywords:** Drug development, Drug development

## Abstract

Recent findings identify a cell-death mechanism in human cancer cells, based on the inhibition of the post-translational modification of NuMA (nuclear mitotic apparatus protein) in cancer cells, which interferes with its protein-binding capacity. NuMA is an indispensable protein for mitosis in both malignant and healthy cells. However, in this cell-death mechanism, only malignant cells are eradicated, due to structural faults inserted in the mitotic spindle poles, causing mitosis arrest. Cell death is imposed in the cancer cells by mitosis arrest, disregarding their mutations.

NuMA is an abundant nuclear protein, changing its subcellular localization and activity throughout the cell-cycle [1–4]. The consensus DNA-binding sites in NuMA and its binding to the cell membrane and to tubulin, implicate NuMA in diverse processes [1–4]. In the interphase, NuMA binding to the DNA is implicated in chromatin organization, including DNA anchorage to the nuclear matrix in the MARS (matrix attached regions) [1], and in chromatin modulation exposing DNA regions to transcription or repair [1]. In the pro-metaphase, the binding of NuMA to tubulin, both directly and indirectly, via the motor protein dynein, implicates NuMA in the construction of the mitotic spindle poles [1–3]. NuMA bound to dynein is also implicated in the binding of NuMA to the cell membrane, the cortical localization of NuMA [1–4]. NuMA is manipulated along the cell-cycle between spindle localization and cortical localization by cyclin-B dependent CDK phosphorylation in specific domains of NuMA [1–4]. NuMA binding to the cell membrane following down-regulation of cyclin-B in the anaphase, is implicated in the separation of the duplicated chromosomes into two equal sets of chromosomes incorporated in the two ‘daughter cells’ [1–4]. In the metaphase, NuMA is implicated in the generation of stable spindle poles [5–7]. This involves the ‘sliding’ of NuMA on the microtubules towards the spindle poles, and its bi-polar clustering while binding to proteins in the poles [5–7]. Stable poles are necessary for the alignment of the duplicated chromosomes in the spindle mid-zone in the anaphase [1–4, 8, 9]. Once all the duplicated chromosomes are aligned in the spindle mid-zone with their kinetochores attached to the microtubules, ubiquitination and degradation of cyclin**-** B by the SAC (spindle assembly control) mechanism leads to NuMA cortical localization and mitosis completion [10]. Un-attachment of scattered chromosomes to the microtubules prevents the completion of mitosis by preventing the ubiquitination of cyclin-B, which causes mitosis arrest in the anaphase [10]. Cell death follows persistent mitosis arrest [10].

NuMA ‘sliding’ towards the spindle poles is dependent on the interaction of NuMA with αβ-tubulin, and this depends on the post-translational modification (PTM) of NuMA by serine-threonine phosphorylation of p34^cdc2^ phosphorylation sites in the globular domain of the carboxy-terminal of NuMA [1, 2, 6, 7]. In addition, in cancer cells, the protein-binding capacity of NuMA is up-regulated by polyADP-ribosylation [11–13]. NuMA is polyADP-ribosylated by the ADP-ribose polymerase, tankyrase1 [11–13]. Tankyrae1 accumulates with NuMA in the spindle poles of cancer cells [11–13]. The binding of proteins in the spindle poles to the ankyrin repeats of tankyrase1 contributes to their stable structure [11–14].

Recent findings identified an exclusive eradication of cancer cells treated with the modified phenanthridine PJ34 ([15] and Table S1 (supplementary data)). PJ34 is generally used for PARP1 inhibition [16]. However, neither PARP1 nor PARP1 inhibition are implicated in this cell death mechanism [17–22]. Furthermore, PJ34 eradicates cancer cells at much higher concentrations than those inhibiting the activity of PARP1 (IC_50_ = 20 nM) [16] (Fig. 1).

**Fig. 1:**
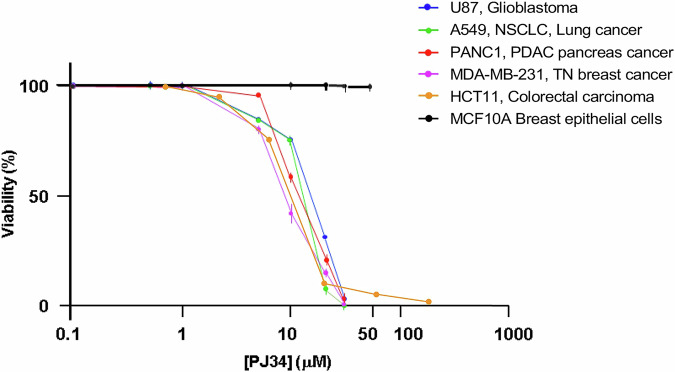
PJ34 eradicates human malignant epithelial cells. An exclusive eradication of the indicated human malignant epithelial cells, treated with PJ34 (96 h) at the indicated concentrations. Benign human epithelial cells are not impaired. From ref. [19].

The eradication of cancer cells is attributable to the interference of PJ34 with the PTM of NuMA in cancer cells, which is essential for NuMA clustering in the spindle poles [15, 18]. PJ34 does not similarly affect the PTM of NuMA in healthy proliferating cells, and these cells continue to proliferate in the presence of PJ34 as untreated cells [18, 20–22].

## Unclustering of NuMA in the spindle poles attributed to PJ34 interference with the PTM of NuMA

**Fig. 2:**
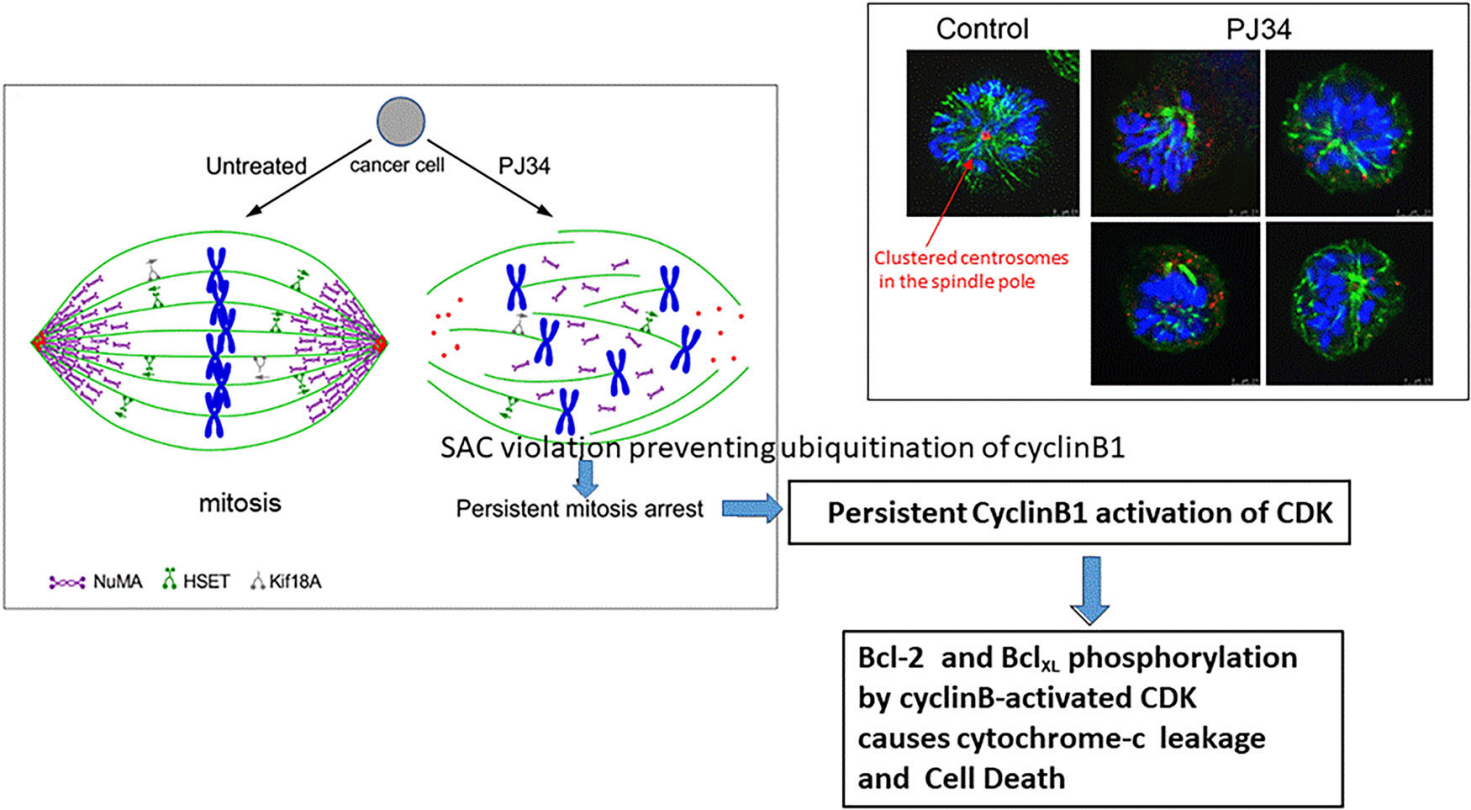
Dispersion of NuMA and duplicated chromosomes in mitotic spindles of multi-centrosomal cancer cells. **Left panel:** A schematic presentation of a normal spindle of an untreated cancer cell in mitosis (control), and an aberrant spindle of PJ34-treated cancer cell. In the treated cancer cell, un-clustered NuMA in the spindle poles, dispersed centrosomes and dispersed duplicated chromosomes, leading to activation of the spindle assembly control (SAC) mechanism, mitosis arrest and cell death. Microtubules (**green**), centrosomes (**red**), NuMA (**purple**) and duplicated chromosomes (**blue**) are displayed. **Right panel:** multi-centrosomal MDA-MB-231 cells. Untreated cell (control): A confocal image of clustered centrosomes (immune-labeled, **red**) in the pole of a well-constructed spindle, and chromosomes aligned in the spindle mid-zone (DAPI-labeling, **blue**). PJ34 treated cells: confocal images of aberrant spindle poles with scattered chromosomes (DAPI-labeling, **blue**) un-aligned in the spindle mid-zone, and dispersed centrosomes (immuno-labeled, **red**). Microtubules are labeled by Immuno-labeled α-tubulin (**green**). From: [15] and [18].

PJ34 eradicates a variety of cancer cells, including cells that are less responsive to current therapies ([15], Fig. 1). Flow-cytometry measurements disclosed G2/M arrest preceding cell death in the PJ34-treated cancer cells [20], while the cell-cycle of PJ34-treated healthy cells was not affected. Healthy cells included epithelial, mesenchymal, and endothelial cells [20, 21]. Concomitantly, structural anomalies were observed in the spindles of cancer cells treated with PJ34, not necessarily multi-centrosomal cancer cells [18, 20–22]. Multiple badly-constructed spindle poles were observed by confocal imaging in PJ34-treated cancer cells [18]. NuMA and tankyrase1 were dispersed in patches in these spindles [18]. Extra centrosomes were also dispersed in the spindles of multi-centrosomal cancer cells [18, 20–22]. Duplicated chromosomes were scattered in the spindles of the treated cancer cells [18, 20–22], and these cells were eradicated within 96 h in cell cultures [18, 20–22] (Figs. 1, 2).

In one of the attempts to identify the molecular mechanism underlying this exclusive cell-death in human cancer cells, the effect of PJ34 on the PTM of proteins that are implicated in mitosis of cancer cells was measured, and compared to the effect of PJ34 on the PTM of the same proteins in healthy proliferating cells [18]. Changes in the PTM of the proteins in four types of epithelial cancer cells and healthy epithelial cells were measured by the shift in their isoelectric point in 2-D gel electrophoresis [18]. The examined malignant epithelial cells included pancreas ductal adenocarcinoma PANC1, breast triple-negative epithelial adenocarcinoma MDA-MB-231, lung alveolarbasalepithelialcells A549, and neuro-epithelial cancer cells, glioblastoma U87 [18].

Out of thirty tested proteins, we identified substantial changes in the isoelectric point of only three proteins in the PJ34-treated epithelial malignant cells versus their isoelectric point in healthy epithelial cells treated with PJ34 [18]. Their isoelectric point was shifted towards higher pH values [18]. These proteins included two kinesins, HSET/kifC1 [23, 24] and kif18A [25], and NuMA [1–4]. These proteins are implicated in the construction of the mitotic spindle. The kinesin HSET/kifC1 is mainly implicated in the construction of the microtubules from building blocks of α-and β-tubulin polymers [23]. The kinesin kif18A is mainly implicated in the binding of microtubules to the duplicated chromosomes in the spindle mid-zone [25]. The bipolar clustering of NuMA is implicated in the construction and stabilization of the spindle poles [1–3, 6, 8, 9]. The stability of the poles is essential for a stable spindle structure and for the alignment of the duplicated chromosomes in the spindle mid-zone [8, 9]. Kinesins HSET/kifC1 and kif18A have already been examined for their possible role in cancer therapy [26], while to the best of our knowledge, NuMA clustering in the spindle poles has not been examined before as a target for cancer cells’ eradication.

The bi-polar clustering of NuMA depends on NuMA interaction with αβ-tubulin [1–3, 5–8, 18], and on the binding of NuMA to proteins in the spindle poles [11–13]. Thus, evidence indicating the dependence of the protein-binding capacity of NuMA on its PTM [7, 11, 18] raised the idea of a possible interference with the PTM of NuMA, preventing the alignment of the duplicated chromosomes in the spindle mid-zone [18].

In cancer cells treated with PJ34, NuMA was not clustered in the spindle poles, and NuMA dispersion was accompanied by scattered duplicated chromosomes in the anaphase [18, 22] (Fig. 2). Also, supernumerary centrosomes were dispersed in the spindles of multi-centrosomal cancer cells treated with PJ34 [18, 20, 22]. Dispersed extra-centrosomes co-localized with dispersed tankyrase1 in PJ34-treated multi-centrosomal cancer cells, suggesting a causal relation between NuMA unclustering in the poles and dispersed extra-centrosomes in poorly constructed multi-polar spindles [18]. None of these structural anomalies was observed in the spindles of PJ34-treated healthy epithelial cells [18, 22].

Cancer cells were eradicated in xenografts as well [18, 27]. A daily intravenous application of PJ34 (50 mg/kg) during 15 days reduced about 90% of PANC1 cells in tumors developed in nude mice, as measured by immuno-histochemistry, 30 days after the treatment with PJ34 was terminated [27]. In contrast, fibroblasts in the mouse origin stroma of the developed PANC1 tumors were not eradicated [27]. The same treatment with PJ34 significantly attenuated tumor growth in nude mice implanted with triple-negative breast cancer cells MDA-MB-232 [18]. PJ34 did not cause any visible adverse effects in the treated mice, and their weight gain was not impaired [27]. In addition, mutations in patients’ derived pancreatic cancer cells [27] and mutations in triple negative breast cancer cells [28] did not affect their eradication by PJ34 [27, 28].

In the PJ34-treated cancer cells, the unclustering of NuMA in spindle poles was accompanied by the interference of PJ34 with NuMA binding to α-tubulin and to kinesins HSET/KifC1 and Kif18A that interact with αβ-tubulin [18]. In addition, PJ34 inhibits the polyADP-ribosylation of NuMA by inhibiting the activity of tankyrase1 [18]. These observations outline a mechanism in which inhibition of the post-translational modifications of NuMA interferes with the protein binding capacity of NuMA, including interference with NuMA sliding on αβ-tubulin towards the spindle poles, and interference with NuMA binding to proteins in the spindle poles [7, 11, 12, 18].

The isoelectric point of NuMA in PJ34-treated epithelial cancer cells was shifted towards higher pH values, in accordance with the prevention of phosphorylation and polyADP-ribosylation of NuMA, causing covalent modifications adding negative charges to modified NuMA [18]. In cancer cells, NuMA is phosphorylated by the serine-threonine kinase pim1 [29–31]. Pim kinases are hardly expressed in healthy cells, while their expression is extensively upregulated in human malignant cells [31]. PJ34 inhibits pim1 kinase (IC_50_ = 3.7 μM) [32]. PJ34 also inhibits the ADP-ribose donor of NuMA, tankyrase1 (IC_50_ = 1. μM, [33]), and tankyrase1 is not expressed in human healthy somatic cells [11–14]. Thus, PJ34 inhibits both tankyrase1 and pim1 kinase, in the range of concentrations inhibiting the PTM of NuMA in cancer cells [18] and eradicates human cancer cells ([18], Fig. 1). This inhibition of the PTM of NuMA could interfere with the assembly of duplicated chromosomes in the spindle mid-zone by preventing the bi-polar clustering of NuMA and tankyrase1 in spindles of PJ34-treated cancer cells [5–9, 11–14]. The resulting unalignment of the duplicated chromosomes in the spindle mid-zone [8, 9, 18] activates the SAC mechanism, which arrests abnormal mitosis before completion in the anaphase by preventing the ubiquitination of cyclin-B [10]. Its expression, as long as mitosis is not completed, results in a persistent activation of cyclin-B-dependent CDK, promoting CDK-catalyzed phosphorylation of several target proteins, including Bcl proteins in the mitochondria [10]. Phosphorylation of Bcl proteins turns the mitochondrial membrane leaky to cytochrome-c [10]. Cytochrome-c release, causing cell death [34], may underlie the eradication of PJ34-treated cancer cells during mitosis arrest [18, 22]. Thus, this cell-death mechanism is induced by an exclusive interference with the PTM of NuMA in cancer cells, inhibiting NuMA polyADP-ribosylation and NuMA phosphorylation by pim1 kinase [18]. This mechanism can cause cell death by mitosis arrest in malignant cells, while mitosis in healthy cells is not impaired [18, 20–22].

Cancer treatments based on cell death during mitosis (Mitotic Catastrophe cell death) have been examined before [35]. Out of several examined mechanisms, only microtubule-targeting inhibitors, taxol and vinca alkaloids, are currently used for cancer therapy, despite their side effects on both healthy proliferating and quiescent cells [35]. Thus, identifying an exclusive eradication of cancer cells during mitosis, which saves the proliferation of healthy cells, could lead to the development of a new cancer therapy. The small molecule PJ34 has many virtues [15, 18]. However, the development of more potent inhibitors targeting the PTM of NuMA in cancer cells might help in translating this cell death mechanism into an effective cancer therapy.

In conclusion, the exclusive PTM of NuMA in cancer cells by polyADP-ribosylation and by Pim1 phosphorylation unveils a new target for the eradication of cancer cells, exploiting the dependence of the bipolar clustering of NuMA in mitotic spindles on its PTM. NuMA bipolar clustering has a major role in the construction of stable spindle poles required for the alignment of the duplicated chromosomes in the spindle mid-zone. Scattered duplicated chromosomes cause mitosis arrest and Mitotic Catastrophe cell death in the anaphase. Thus, molecules exclusively interfering with the PTM of NuMA in cancer cells can kill cancer cells while retaining the proliferation of healthy cells.

## Supplementary information

Table S1. Types of cancer cells that are eradicated by PJ34

## References

[1] Kiyomitsu T, Boerner S. The Nuclear Mitotic Apparatus (NuMA) Protein: A key player for nuclear formation, spindle assembly, and spindle positioning. Front Cell Dev Biol. 2021;9:653801. 33869212 10.3389/fcell.2021.653801 PMC8047419

[2] Zheng Z, Wan Q, Meixiong G, Du Q. Cell cycle-regulated membrane binding of NuMA contributes to efficient anaphase chromosome separation. Mol Biol Cell. 2014;25:606–19. 24371089 10.1091/mbc.E13-08-0474 PMC3937087

[3] Radulescu AE, Cleveland DW. NuMA after 30 years: the matrix revisited. Trends Cell Biol. 2010;20:214–22. 20137953 10.1016/j.tcb.2010.01.003 PMC3137513

[4] Kotak S, Busso C, Gönczy P. NuMA phosphorylation by CDK1 couples mitotic progression with cortical dynein function. EMBO J. 2013;32:2517–29. 23921553 10.1038/emboj.2013.172 PMC3770949

[5] Haren L. Andreas-Merdes. Direct binding of NuMA to tubulin is mediated by a novel sequence motif in the tail domain that bundles and stabilize microtubules. J Cell Sci. 2002;115:1815–23. 11956313 10.1242/jcs.115.9.1815

[6] Silk AD, Holland AJ, Cleveland DW. Requirement for NuMA in maintenance and establishment of mammalian spindle poles. J Cell Biol. 2009;184:677–90. 19255246 10.1083/jcb.200810091 PMC2686415

[7] Compton DA, Luo C. Mutations in the predicted p34^cdc2^ phosphorylation sites in NuMA impair the assembly of the mitotic spindle and block mitosis. J Cell Sci. 1995;108:621–33. 7769006 10.1242/jcs.108.2.621

[8] Haren L, Gnadt N, Wright M, Merdes A. NuMA is required for proper spindle assembly and chromosome alignment in prometaphase. BMC Res Notes. 2009;2:64. 19400937 10.1186/1756-0500-2-64 PMC2686716

[9] Gordon MB, Howard L, Compton DA. Chromosome movement in mitosis requires microtubules anchorage at spindle poles. J Cell Biol. 2001;152:425–34. 11157972 10.1083/jcb.152.3.425 PMC2196006

[10] Lara-Gonzales P, Westhorpe FG, Taylor SSA. spindle assembly checkpoint. Curr Biol. 2012;22:R966–80. 23174302 10.1016/j.cub.2012.10.006

[11] Chang P, Coughlin M, Mitchison J. Interaction between polyADP-ribose and NuMA contributes to mitotic spindle pole assembly. Mol Biol Cell. 2009;20:4575–85. 19759176 10.1091/mbc.E09-06-0477 PMC2770945

[12] Chang P, Coughlin M, Mitchelson TJ. Tankyrase-1 polymerization of poly(ADP-ribose) is required for spindle structure and function. Nat Cell Biol. 2005;7:1133–9. 16244666 10.1038/ncb1322

[13] Lehtio L, Chi N-W, Krauss S. Tankyrases as drug targets. FEBS J. 2013;280:3576–93. 23648170 10.1111/febs.12320

[14] Eisemann T, McCauley M, Langelier M-F, Gupta K, Roy S, Van Duyne DG, et al. Tankyrase-1 Ankyrin repeats form an adaptable binding platform for targets of ADP-Ribose modification. Structure. 2016;24:1679–92. 27594684 10.1016/j.str.2016.07.014

[15] Cohen-Armon M. The modified phenanthridine PJ34 unveils an exclusive cell-death mechanism in human cancer cells. Cancers. 2020;12:1628. 32575437 10.3390/cancers12061628 PMC7352794

[16] Abdelkarim GE, Gertz K, Harm C, Katchanov J, Dirnagi U, Szabo C, et. al. Protective effect of PJ34, a novel potent inhibitor of poly(ADP-ribose) polymerase (PARP) in in-vitro and in-vivo models of stroke. Int J Mol Med. 2001;7:255–60. 11179503

[17] Madison DL, Stauffer D, Lundblad JR. The PARP inhibitor PJ34 causes a PARP1-independent, p21 dependent mitotic arrest. DNA Repair. 2011;10:1003–13. 21840268 10.1016/j.dnarep.2011.07.006 PMC3185120

[18] Visochek L, Castiel A, Mittelman L, Elkin M, Atias D, Golan T, et al. Exclusive destruction of mitotic spindles in human cancer cells. Oncotarget. 2017;8:20813–24. 28209915 10.18632/oncotarget.15343 PMC5400547

[19] Cohen-Armon M. Exclusive modifications of NuMA in malignant epithelial cells: A potential therapeutic mechanism. Drug Discov Today. 2022;27:1205–09. 35143964 10.1016/j.drudis.2022.02.002

[20] Castiel A, Visochek L, Mittelman L, Dantzer F, Izraeli S, Cohen-Armon M. A phenanthrene derived PARP inhibitor is an extra-centrosomes de-clustering agent exclusively eradicating human cancer cells. BMC Cancer. 2011;11:412. 21943092 10.1186/1471-2407-11-412 PMC3204305

[21] Inbar-Rozensal D, Visochek L, Castel D, Castiel A, Izraeli S, Dantzer F, et al. A selective eradication of human nonhereditary breast cancer cells by phenanthridine-derived polyADP-ribose polymerase inhibitors. Breast Cancer Res. 2009;11:R78. 19891779 10.1186/bcr2445 PMC2815540

[22] Castiel AG, Visochek L, Mittelman L, Zilberstein Y, Dantzer F, Izraeli S, et al. Cell-death associated with abnormal mitosis observed by confocal imaging in live cancer cells. JoVE. 2013;78:e50568. 10.3791/50568 PMC3856276 23995751

[23] Hirokawa N, Noda Y, Tanaka Y, Niwa S. Kinesin superfamily motor proteins and intracellular transport. Nat Rev Mol Cell Biol. 2009;10:682–96. 19773780 10.1038/nrm2774

[24] Verhey K, Hammond JW. Traffic control: regulation of kinesin motors. Nat Rev Mol Cell Biol. 2009;10:765–77. 19851335 10.1038/nrm2782

[25] Mayr MI, Hummer S, Bormann J, Gruner T, Adio S, Woehlke G, et al. The human kinesin Kif18A is a motile microtubule depolymerase essential for chromosome congression. Curr Biol. 2007;17:488–98. 17346968 10.1016/j.cub.2007.02.036

[26] Liu X, Gong H, Huang K. Oncogenic role of kinesin proteins and targeting kinesin therapy. Cancer Sci. 2013;104:651–6. 23438337 10.1111/cas.12138 PMC7657121

[27] Visochek L, Atias D, Spektor I, Castiel A, Golan T, Cohen-Armon M. The phenanthridine derivative PJ34 exclusively eradicates human pancreatic cancer cells in xenografts. Oncotarget. 2019;10:6269–82. 31692907 10.18632/oncotarget.27268 PMC6817443

[28] Keung MY, Wu Y, Badar F, Vadgama JV. Response of breast cancer cells to PARP inhibitors is independent of BRCA status. J Clin Med. 2020;9:940. 32235451 10.3390/jcm9040940 PMC7231148

[29] Bhattacharya N, Wang Z, Davitt C, McKenzie IF, Xing PX, Magnuson NS. Pim-1 associates with protein complexes necessary for mitosis. Chromosoma. 2002;111:80–95. 12111331 10.1007/s00412-002-0192-6

[30] Mondello P, Cuzzocrea S, Mian M. Pim kinases in hematological malignancies: where are we now and where are we going?. Rev J Hematol. 2014;7:95–104. 10.1186/s13045-014-0095-z PMC4266197 25491234

[31] Walhekar V, Bagul C, Kuma D, Muthal A, Achaiah G, Ulkarni RK. Topical advances in PIM kinases and their inhibitors: Medicinal chemistry perspectives. Biochim Biophys Acta Rev Cancer. 2022;1877:188725. 35367531 10.1016/j.bbcan.2022.188725

[32] Antolín AA, Jalencas X, Yelamos J, Mestres J. Identification of Pim kinases as novel targets for PJ34 with confounding effects in PARP biology. ACS Chem Biol. 2012;7:1962–7. 23025350 10.1021/cb300317y

[33] Kirby CA, Cheung A, Fazal A, Shultz MD, Stam T. Structure of human tankyrase1 in complex with small molecule inhibitors PJ34 and XAV939. Acta Crystallogr. 2012;F68:115–8. 10.1107/S1744309111051219 PMC3274384 22297980

[34] Garrido C, Galluzzi L, Brunet M, Puig PE, Didelot C, Kroemer G. Mechanisms of cytochrome *c* release from mitochondria. Cell Death Differ. 2006;13:1423–33. 16676004 10.1038/sj.cdd.4401950

[35] Mc Gee MM. Targeting the mitotic catastrophe signaling pathway in cancer. Mediators Inflamm. 2015;2015:146282. 26491220 10.1155/2015/146282 PMC4600505

