# Supplementary material for: The post-translational modification of NuMA in cancer cells is a new target for cancer eradication
Source: Cell Death Dis. 2025 Jul 18;16(1):536. doi: 10.1038/s41419-025-07868-7 (PMC12274338; doi:10.1038/s41419-025-07868-7)
Supplement: Supplementary file 1 — Table S1. Types of cancer cells that are eradicated by PJ34 [file 41419_2025_7868_MOESM1_ESM.docx]

**Supplementary Data**

**Table S1. Types of cancer cells that are eradicated by PJ34**

| **Cancer type** | **Reference** |
| --- | --- |
| Breast cancer: MDA-MB-436, MDA-MB468, HCC1937, BT549, HCC1143, HCC1806, SKBR, JIMT, MCF7, BT47 | 1 |
| Breast cancer: triple negative MDA-MB-231, MCF7 | 2,3,4 |
| Colon cancer DLD1 | 5 |
| Colorectal HCT11 | 5 |
| Ovary HeyA8 | 5 |
| Lung H1299, A549 | 3, 5 |
| Lung Calu-6, A549, H460 | 6 |
| Pancrease PANC1 | 3, 7 |
| Melanoma M14 | 8 |
| Multiple Myeloma PPM18226 | 9 |
| Liver HepG2, SMMC7721 | 10 |
| Glioblastoma U87 | 3,11 |
| Leukemia cell lines ATLL, transformed HTLV-I | 12 |
| HeLa cells | 13 |
| Thyroid cancer cell lines ( TPC1,BCPAP,WRO,FRO ) | 14 |

**References**

1. Keung MY, Wu Y, Badar F, Vadgama JV. Response of breast cancer cells to PARP inhibitors is independent of BRCA status. *J. Clin. Med.*  **9**, 940 (2020)

2 Inbar-Rozensal D, Visochek L, Castel D, Castiel A, Izraeli S, Dantzer F, et al. Selective eradication of human nonhereditary breast cancer cells by phenanthridine-derived polyADP-ribose polymerase inhibitors. *Breast Cancer Res* **11**, R78 (2009)

3 Visochek L, Castiel A, Mittelman L, Elkin M, Atias D, Golan T, et al. Exclusive destruction of mitotic spindles in human cancer cells. *Oncotarget* **8**, 20813-20824 (2017)

4 Castiel A, Visochek L, Mittelman L, Zilberstein Y, Dantzer F, Izraeli S, et al. Cell-death associated with abnormal mitosis observed by confocal imaging in live cancer cells *JoVE* **78**, e50568 (2013)

5 Castiel A, Visochek L, Mittelman L, Dantzer F, Izraeli S, Cohen-Armon, M. A phenanthrene derived PARP inhibitor is an extra-centrosomes de-clustering agent exclusively eradicating human cancer cells. *BMC Cancer* **11**, 412 (2011)

6. Gangopadhyay NN, Luketich JD, Opest A, Meyer EM, Landreneau R, Schuchert MJ. Inhibition of Poly(ADP-Ribose) Polymerase (PARP) Induces Apoptosis in Lung Cancer Cell Lines. *Cancer Investig.*  **29**, 608–616 (2011)

7. Visochek L, Atias D, Spektor I, Castiel A, Golan T, Cohen-Armon M. The phenanthridine derivative PJ34 exclusively eradicates human pancreatic cancer cells in xenografts. *Oncotarget* **10**,6269-6282 (2019)

8. Chevanne M, Zampieri M, Rizzo A.C.R, Ciccarone F, Catizone A, D’Angelo C, et al. Inhibition of PARP activity by PJ34 leads to growth impairment and cell death associated with aberrant mitotic pattern and nucleolar actin accumulation in M14 melanoma cell line. *J. Cell Physiol.*  **222**, 401–410 (2010).

9. Xiong T, Chen X, Wei H, Xiao H. Influence of PJ34 on the genotoxicity induced by melphalan in human multiple myeloma cells. *Arch. Med. Sci.*  **11**, 301–306 (2015)

10. Liang B, Xiong M, Ji G, Zhang E, Zhang Z, Dong K, et al. Synergistic suppressive effect of PARP-1 inhibitor PJ34 and HDAC inhibitor SAHA on proliferation of liver cancer cells. *J. Huazhong Univ. Sci. Technol.*  **35**, 535–540 (2015)

11. Majuelos-Melguizo J, Rodríguez MI, López-Jiménez L, Jose M, Rodríguez-Vargas J, Martín-Consuegra M, et al. PARP targeting counteracts gliomagenesis through induction of mitotic catastrophe and aggravation of deficiency in homologous recombination in PTEN-mutant glioma. *Oncotarget* **6**, 4790–4803 (2015)

12. Bai XT, Moles R, Chaib-Mezrag H, Nicot C. Small PARP inhibitor PJ34 induces cell-cycle arrest and apoptosis of adult T cell leukemia cells. *J. Hematol. Oncol.*  **8**, 117 (2015)

13. Magan N, Isaacs RJ, Stowell KM. Treatment with the PARP-inhibitor PJ34 causes enhanced doxorubicin-mediated cell death in HeLa cells. *Anticancer Drugs* **23**, 627–637 (2012)

14. Lavarone E, Puppi C, Passon N, Filetti S, Russo D, Damante G. The PARP inhibitor PJ34 modifies proliferation, NIS expression and epigenetic marks in thyroid cancer cell lines. *Mol. Cell Endocrinol.*  **365**, 1–10 (2013).
